# Treatment of patients with borderline personality disorder in the emergency room? A scoping review

**DOI:** 10.3389/fpsyt.2026.1820717

**Published:** 2026-07-08

**Authors:** Marissa Bouchard-Boivin, Alexandre Hudon, Francis Godin, Marie Désilets, Lionel Cailhol

**Affiliations:** 1 Department of Psychiatry and Addictology, Faculty of Medicine, Université de Montréal, Montreal, QC, Canada; 2 Department of Psychiatry, Institut universitaire en santé mentale de Montréal, Université de Montréal, Montreal, QC, Canada; 3 Centre de recherche de l’Institut universitaire en santé mentale de Montréal, Montreal, QC, Canada; 4 Department of Psychiatry, Institut national de psychiatrie légale Philippe-Pinel, Montreal, QC, Canada; 5 Centre de recherche en pédagogie de la santé, Université de Montréal, Montreal, QC, Canada; 6 Service de psychiatrie, Hôpital de la Cité-de-la-Santé, Laval, QC, Canada

**Keywords:** acute psychiatric management, borderline personality disorder, brief hospitalization, crisis intervention, emergency department, service pathways, suicide risk assessment

## Abstract

**Background:**

Borderline personality disorder (BPD) is frequently encountered in emergency departments. Acute emotional crises, suicidal behaviors, and severe interpersonal distress often precipitate care. Despite the high clinical burden and elevated suicide risk associated with this disorder, guidance for emergency decision-making remains fragmented and sometimes contradictory.

**Objective:**

This scoping review aimed to identify key elements informing emergency psychiatric assessment, disposition decisions, and acute management strategies for adults diagnosed with BPD. Methods: Following Preferred Reporting Items for Systematic Reviews and Meta-Analyses extension for Scoping Reviews guidelines, four electronic databases were searched for studies published between January 1^st,^ 2000, and December 31^st^, 2025, examining evaluation, orientation, or management of adults with BPD in emergency settings. Eligible studies were screened independently, and data were charted and synthesized thematically.

**Results:**

Twelve studies met inclusion criteria. Three interrelated domains emerged: structured suicide risk assessment contextualized within chronic vulnerability; preference for brief, goal-directed crisis hospitalization over prolonged inpatient admission; and cautious, symptom-targeted pharmacological use limited primarily to short-term management of acute agitation. Evidence consistently highlighted a paradox in which individuals with BPD often present with severe distress but are admitted less frequently than other psychiatric populations. Emerging clinical pathways emphasize voluntary short stays, rapid follow-up, and linkage to outpatient services to reduce recurrent emergency presentations and iatrogenic harm. Overall, pharmacotherapy plays an adjunctive rather than a central role in emergency care.

**Conclusions:**

Emergency encounters with individuals living with BPD represent critical inflection points in trajectories often marked by recurrent crises and suicide risk. Current evidence supports structured, formulation-based assessment, time-limited crisis stabilization, and integration with outpatient services, while underscoring significant gaps in high-quality research. Strengthening system-level pathways may enhance safety, continuity, and therapeutic engagement in this high-risk population.

## 1 Introduction

Borderline personality disorder (BPD) is characterized by pervasive instability in affect regulation, interpersonal relationships, and self-image, accompanied by marked impulsivity and recurrent self-destructive behaviors, including suicidal acts (1). A core feature of the disorder is profound sensitivity to real or perceived abandonment, which often precipitates intense emotional dysregulation and maladaptive coping strategies. Individuals with BPD also frequently experience chronic feelings of emptiness, intense anxiety, and unstable self-perception, which may further contribute to crisis presentations and recurrent emergency service utilization. With an estimated lifetime prevalence of 1%-2% in the general population, BPD is a major public health concern (2). Its high rates of psychiatric comorbidity (including mood, anxiety, substance use, and post-traumatic stress disorders as well as the frequency of self-injurious and suicidal behaviors, contribute to substantial functional impairment and recurrent utilization of emergency services (3–7).

Accordingly, personality disorders account for a significant proportion of psychiatric emergency consultations. Prevalence rates in psychiatric emergency departments have been reported as high as 26%, with BPD alone representing approximately 9% of visits (8, 9). The burden extends beyond psychiatric facilities: in general hospitals, BPD is identified in nearly 2% of all admissions, underscoring its cross-sector impact on healthcare systems (10).

Emergency department (ED) presentations frequently result in either psychiatric hospitalization or referral to outpatient services. However, a striking paradox has emerged in the literature: although individuals with BPD presenting to emergency services often exhibit greater clinical severity compared to other psychiatric populations, they are less likely to be admitted to inpatient units (11–13). Multiple factors may contribute to this discrepancy. Emotional dysregulation and interpersonal volatility may complicate engagement during acute assessment. Structural stigma and therapeutic pessimism toward personality disorders may also influence clinical decision-making. Furthermore, clinical guidelines often emphasize outpatient psychotherapeutic management as the preferred intervention, but access to evidence-based ambulatory treatments is inconsistent or insufficient in many settings (10, 14).

This issue is particularly concerning, considering suicide risk data. An Australian study found that approximately one quarter of individuals with BPD who died by suicide had presented to an emergency department within the six weeks preceding their death (15). Clinicians are therefore confronted with a complex and high-stakes dilemma: whether to proceed with hospitalization (an intervention associated in some reports with potential symptom aggravation and limited protective effect against suicide risk) or to discharge patients toward outpatient services whose availability and continuity of care may be uncertain (16–18). The ethical and clinical tensions are substantial, involving both the imperative to ensure patient safety and the responsibility to direct individuals toward interventions with demonstrated efficacy.

Through a scoping review, we sought to identify key elements to inform clinical decision-making during emergency psychiatric assessments of individuals diagnosed with BPD. Specifically, our objectives were: (1) to delineate factors relevant to hospitalization versus discharge decisions; (2) to clarify recommended care pathways following emergency evaluation; and (3) to synthesize available evidence regarding pharmacological interventions applicable to this population in acute settings.

## 2 Methods

### 2.1 Search strategies

This scoping review was conducted in accordance with the Preferred Reporting Items for Systematic Reviews and Meta-Analyses extension for Scoping Reviews (PRISMA-ScR) (19). A comprehensive and systematic search strategy was developed to identify empirical and review literature examining the evaluation, orientation, and management of patients diagnosed with BPD in emergency settings.

The electronic databases PubMed, Embase, PsycInfo, and CINAHL were searched to ensure multidisciplinary coverage of psychiatric, medical, psychological, and nursing literature. The search strategy combined controlled vocabulary terms (e.g., MeSH, Emtree) and free-text keywords to maximize sensitivity. Core search concepts included “personality disorder(s),” “borderline AND personality,” and “emergency.” Boolean operators were used to combine terms, and truncation and proximity operators were applied where appropriate to capture variations in terminology. The full search strategy for each database, including the syntax and applied limits, is provided in the Supplemental Materials.

The search covered publications from January 1, 2000, to December 31, 2025. This timeframe was selected to reflect contemporary conceptualizations of BPD, developments in emergency psychiatric practice, and evolving clinical guidelines regarding hospitalization and outpatient management. No restrictions were imposed at the search stage on study design to capture the breadth of available evidence relevant to clinical decision-making in emergency departments. The search strategy was developed collaboratively by MBB and MD, both experienced in psychiatric research methodology.

### 2.2 Studies eligibility

Eligibility criteria were predefined to ensure conceptual coherence with the objectives of the review while maintaining the exploratory scope consistent with scoping review methodology.

Included studies were required to be published in either French or English and conducted in Western countries. This restriction was implemented to maximize comparability across healthcare systems, emergency psychiatric service organization, and medico-legal frameworks governing hospitalization and suicide-risk management. Given the exploratory objectives of this review, greater contextual homogeneity was considered preferable to broader geographic coverage. The population of interest consisted of adults aged 18 to 65 years. Studies focusing exclusively on adolescents or geriatric populations were excluded due to distinct clinical and service considerations in those age groups.

Eligible articles had to involve patients whose primary diagnosis established in the emergency department was clinical BPD. Studies were retained if comorbid psychiatric conditions were present, if BPD was identified as the principal diagnosis guiding the emergency assessment. The diagnostic process had to occur within an emergency setting and be conducted by a psychiatrist or an emergency physician, thereby ensuring clinical validity and relevance to acute care decision-making.

Studies were required to address either the assessment process in emergency settings, clinical orientation (e.g., hospitalization versus discharge), or therapeutic management strategies, including pharmacological approaches, for patients presenting with BPD. Articles focusing solely on long-term outpatient psychotherapy without reference to emergency evaluation were excluded.

The study selection process followed a two-stage screening procedure consistent with PRISMA-ScR recommendations. Titles and abstracts were independently screened by AH and MBB. To minimize the risk of excluding potentially relevant evidence, abstracts mentioning personality disorders more broadly (without explicitly specifying BPD) were retained for full-text review. Full-text screening was subsequently performed independently by MBB, FG, and AH. Discrepancies regarding inclusion were resolved through discussion and consensus, ensuring methodological rigor and reducing selection bias.

When review articles were included, their referenced primary studies were cross-checked against directly included studies to minimize redundancy in interpretation.

### 2.3 Data extraction

Data extraction was conducted using a standardized data-charting form developed specifically for this review. The form was designed to capture bibliographic characteristics, study design, country of origin, sample characteristics, diagnostic procedures, emergency setting characteristics, assessment frameworks, clinical orientation decisions (e.g., hospitalization, referral to outpatient services), and pharmacological or other acute interventions described.

Extraction was performed by MBB to ensure consistency across studies and subsequently reviewed by AH for accuracy and completeness. Any ambiguities in data interpretation were discussed among the research team to ensure consensus and fidelity to the original sources. The data-charting process was iterative, allowing refinement of extraction categories as familiarity with the literature evolved, in line with best practices for scoping reviews.

### 2.4 Data analysis

Given the exploratory aim of identifying key elements relevant to emergency clinical decision-making for patients with BPD, a descriptive and thematic synthesis approach was employed. Quantitative findings were summarized narratively, focusing on patterns related to hospitalization rates, predictors of admission, suicide risk considerations, and pharmacological practices in emergency settings.

Qualitative data and conceptual analyses were synthesized using thematic analysis to identify recurrent domains that influence emergency decision-making. Attention was paid to structural factors (e.g., service availability), clinician-related variables (e.g., perceived risk, stigma, guideline adherence), and patient-related clinical features (e.g., acute suicidality, emotional dysregulation, comorbid substance use).

Consistent with PRISMA-ScR methodology, the objective was not to produce pooled effect estimates but rather to map the scope of existing evidence, identify areas of convergence and divergence, and highlight knowledge gaps relevant to emergency psychiatric practice. Findings were organized to inform three core domains: determinants of hospitalization decisions, recommended post-emergency care pathways, and evidence regarding pharmacological interventions in acute settings.

### 2.5 Quality assessment

Although formal quality appraisal is not mandatory in scoping reviews, a methodological quality assessment was conducted to contextualize the strength and limitations of the available evidence. Systematic reviews included in the sample were evaluated using the AMSTAR tool to assess their methodological quality. Observational studies were appraised using the quality assessment grid developed by the Centre hospitalier universitaire de Québec.

Quality appraisal was independently conducted by MBB and AH. Discrepancies were resolved through discussion and consensus. The purpose of the appraisal was not to exclude studies based on methodological limitations but to inform interpretation of findings and to distinguish between higher- and lower-quality evidence when synthesizing conclusions.

## 3 Results

### 3.1 Description of the identified studies

**Figure 1:**
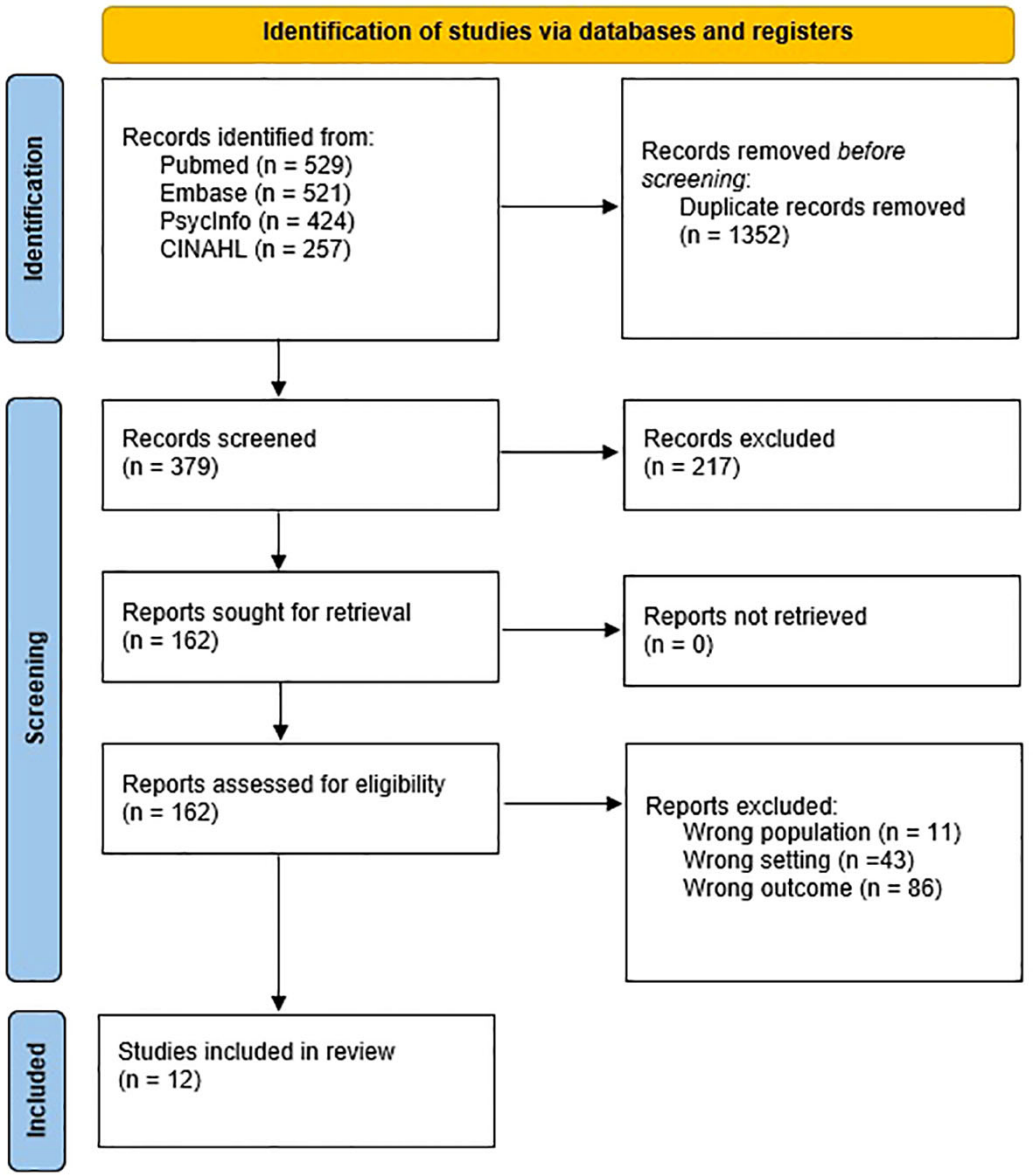
PRISMA flowchart.

The systematic search yielded a total of 1,721 records (see **Figure 1**). Database-specific retrieval included 529 articles from PubMed, 521 from Embase, 424 from PsycInfo, and 257 from CINAHL. Following automated duplicate removal using reference management software, records underwent manual verification to identify residual duplicates across databases. The remaining unique records were then subjected to title and abstract screening according to predefined eligibility criteria. Of these, 217 were excluded at the title-screening and abstract-screening stages because they did not meet the predefined inclusion criteria. A total of 150 articles were subsequently excluded after full-text review because they did not fulfill inclusion and exclusion criteria. Five book chapters were identified but excluded due to methodological ineligibility. Finally, 12 articles met all criteria and were included in the final synthesis.

The retained studies reflected markedly heterogeneous designs, including clinical guidelines, observational studies, retrospective audits, naturalistic investigations, intervention reports, and narrative reviews. This variability in methodological approaches limited direct comparability across studies and reduced the strength of evidence supporting several clinical recommendations. Collectively, they addressed three interrelated domains relevant to emergency psychiatric care for individuals with BPD: suicide risk assessment, orientation decisions including hospitalization strategies, and pharmacological considerations in acute settings.

### 3.2 Main results

Across the included studies, a consistent emphasis emerged on the centrality of structured suicide risk assessment during emergency presentations (20, 21). Beyond immediate risk stratification, authors underscored the importance of contextualizing the acute crisis within the patient’s broader psychosocial and psychiatric history. Emergency encounters were conceptualized not solely as risk-management episodes but as critical clinical junctures requiring formulation of precipitating factors, including interpersonal ruptures, affective instability, substance misuse, and treatment discontinuity (21).

A second major finding concerned the paradoxical nature of hospitalization decisions in BPD. While individuals with BPD frequently present with high levels of distress, suicidality, and emotional dysregulation, the literature cautions against routine or prolonged inpatient admission. Instead, a model favoring structured, time-limited crisis intervention emerged as the most consistently recommended approach (9, 20–22).

Finally, pharmacological management in the emergency setting was characterized by prudence and restraint. The literature generally discouraged initiating long-term pharmacotherapy during acute emergency visits, while recognizing a limited role for targeted short-term pharmacological interventions in the management of acute agitation (9). Details about the identified studies are presented in **Table 1**.

**Table 1:** Summary of the articles selected for the literature review.

Authors	Article type	Population	Objectives/themes	Results/observations
Berrino et al. (23)	Prospective cohort study (3 months)	Patients aged 18–65 with BPD (DSM-IV) referred to ED after deliberate self-harm (n=100)	Evaluate whether crisis intervention is appropriate in ED management	Reduced self-harm behaviors and fewer rehospitalizations at 3 months in crisis intervention group
Biskin & Paris (24)	Literature review	Patients with BPD (33 articles reviewed)	Management of BPD in emergency settings	Hospitalization should generally be avoided; brief hospitalization may be considered in serious suicide attempts or psychotic symptoms; short stays (24h) for minor suicidal behaviors; referral to external teams recommended
Broadbear et al. (10)	Retrospective electronic audit (12 months)	583 confirmed BPD patients presenting to ED (Melbourne) vs depression-only cohort	Describe prevalence, characteristics, and outcomes of ED presentations	BPD accounted for around 2% ED visits but high repetition (mean 4.8/year); high comorbidity; frequent ambulance arrival; repeated presentations; need structured referral pathways
Cailhol et al. (25)	Case-control study (10 months)	Patients admitted after severe medication overdose (n=478; 99 with BPD)	Assess influence of BPD diagnosis on ED orientation decisions	BPD diagnosis did not significantly influence clinical orientation decisions
Cailhol et al. (25)	Exploratory study	Suicidal adults admitted after voluntary overdose (FRENCH CRISIS study; n=320)	Influence of hospitalization on 6-month recurrence in high-risk BPD patients	Hospitalization did not reduce 6-month recurrence; high-risk BPD patients not more frequently hospitalized
Damsa et al. (26)	Prospective observational study	25 agitated BPD patients evaluated with SCID in ED	Efficacy and safety of IM olanzapine	Olanzapine effective for acute agitation with few side effects
Golcuk & Şahin (27)	Living reference work entry (Encyclopedia)	Adults with BPD in emergency settings	Comprehensive framework for ED management including ethical and interpersonal dimensions	Structured suicide risk assessment; validation and de-escalation; trauma-informed care; limited short-term pharmacology; DBT-informed brief approaches; attention to autonomy and ethics
Pascual et al. (28)	Open study	12 BPD patients in psychiatric emergency (9 completed)	Efficacy and safety of ziprasidone for acute agitation	Ziprasidone effective and rapid; few side effects
Pascual et al. (29)	Open-label naturalistic case series	20 BPD patients in psychiatric emergency	Efficacy and safety of atypical antipsychotics in agitation	Ziprasidone 20 mg IM and olanzapine 10 mg IM effective and rapid; few side effects
Pascual et al. (9)	Prospective naturalistic study	Large cohort DSM-IV BPD vs non-BPD patients	Factors associated with hospitalization and psychotropic prescription	Fewer BPD patients hospitalized despite severity; hospitalization linked to suicide risk, danger to others, severe symptoms, impaired self-care, non-adherence
Shaikh et al. (21)	Literature review	Patients with BPD in ED (56 articles)	Guidance for ED management	Partial hospitalization for crisis; atypical antipsychotics effective for agitation; benzodiazepines/typical antipsychotics possible but with side-effect risks
Webb et al. (22)	Clinical pathway proposal (service model)	Patients with BPD presenting to ED in crisis (Australia)	Propose evidence-based acute clinical pathway	Structured ED assessment; brief voluntary admission (≤48h); avoid involuntary admission; 4-session rapid follow-up; linkage to long-term care; reduce iatrogenic harm

### 3.3 Evaluation conducted in the emergency setting

The reviewed studies converge on the necessity of a thorough and multidimensional suicide risk assessment in patients with BPD presenting to emergency departments (20, 21). This evaluation should extend beyond the presence of current suicidal ideation and incorporate a detailed exploration of established risk factors. These include prior suicide attempts, comorbid mood disorders or substance use disorders, elevated levels of hopelessness, a family history of suicidal behavior or completed suicide, a history of sexual abuse, and high levels of impulsivity or antisocial traits.

The assessment process is therefore not limited to actuarial risk scoring but requires clinical judgment informed by both acute symptom severity and longitudinal vulnerability factors. Importantly, several authors emphasize that risk assessment should not overshadow exploration of the immediate precipitating crisis. Understanding the interpersonal, environmental, or treatment-related triggers that led to the emergency consultation is considered essential for determining appropriate orientation and mitigating short-term recurrence (21). This formulation-based approach situates the emergency evaluation within a broader therapeutic trajectory rather than treating it as an isolated event. More recently, structured clinical pathways have been proposed to standardize the emergency assessment of patients with BPD. A 2023 Australian model emphasizes the use of a defined triage algorithm, confirmation of diagnosis when appropriate, structured suicide risk assessment, avoidance of exploratory trauma-focused interviewing during acute crises, and clear documentation of admission goals prior to disposition (22). This pathway explicitly links ED assessment to rapid follow-up interventions to reduce iatrogenic harm and recurrent crisis presentations.

A large retrospective Australian audit of 583 patients with confirmed BPD diagnoses found that although individuals with BPD accounted for approximately 2% of total ED presentations, nearly three-quarters re-presented within the same year, with a mean of almost five visits per patient. Frequent presenters were more likely to arrive by ambulance, exhibit psychiatric comorbidity, and have multiple service involvements (10). Notably, the highest-frequency attenders were paradoxically less likely to be admitted, illustrating the tension between perceived risk, chronicity, and admission practices.

Recent conceptual syntheses of emergency BPD care further emphasize structured suicide risk tools (e.g., Columbia-Suicide Severity Rating Scale), alongside trauma-informed communication strategies, emotional validation, and explicit boundary setting. These frameworks highlight the dual need to ensure safety while preserving patient autonomy in high-intensity emergency environments (27).

### 3.4 Treatment plan in the emergency setting

A recently proposed structured pathway recommends that admissions be voluntary, brief (typically overnight or up to 48 hours), and goal-directed, with clearly defined discharge plans and linkage to a short-term follow-up clinic offering four rapid sessions of crisis-focused intervention (22). Such models aim to prevent overdependence on acute services while maintaining patient safety. With respect to disposition decisions, brief hospitalization (distinct from conventional or prolonged inpatient admission) emerges as the most consistently recommended strategy (9, 10, 20–22). This approach is supported by clinical research demonstrating that a structured five-day crisis hospitalization was associated with a reduction in subsequent hospitalizations and emergency department visits within three months following discharge (23). Such findings suggest that time-limited, goal-oriented inpatient interventions may stabilize acute crises without reinforcing maladaptive dependency dynamics.

Indications for hospitalization include elevated suicide risk, danger to others, severe symptom exacerbation, inability to care for oneself, and significant treatment non-adherence. However, several studies caution against extended inpatient stays, citing potential risks of regression, including increased substance use, behavioral acting out, passivity, and diminished self-efficacy (20, 22). Some authors also suggest that repeated or prolonged hospitalizations may paradoxically contribute to longer-term suicide risk (24). Nevertheless, a French naturalistic study did not identify an increased risk at six months following hospitalization decisions, highlighting ongoing debate and the need for individualized clinical judgment (27).

Regarding pharmacological interventions, most sources recommend avoiding initiating new long-term medication regimens during emergency consultations (9). The emergency context is considered suboptimal for initiating maintenance pharmacotherapy, given the instability of the clinical situation and the limited opportunity for longitudinal monitoring. However, in cases of acute agitation, several studies and clinical observations support the preferential use of second-generation antipsychotics, particularly intramuscular olanzapine and ziprasidone, due to their relatively favorable tolerability profiles and rapid onset of action in emergency settings (21, 26, 28, 29). Olanzapine appears particularly useful when agitation occurs in the context of marked affective dysregulation, whereas ziprasidone has also demonstrated short-term effectiveness in psychiatric emergency services. Benzodiazepines may occasionally be used adjunctively in highly anxious or severely agitated presentations, although caution is warranted due to risks of oversedation, behavioral disinhibition, and respiratory depression, especially when combined with first-generation antipsychotics. Whenever possible, pharmacological interventions should be embedded within verbal de-escalation and trauma-informed approaches rather than used as stand-alone interventions. The combination of a first-generation antipsychotic with a benzodiazepine is discouraged due to the increased risk of adverse effects, including dystonia and respiratory depression (21). Overall, pharmacological management in the emergency setting is conceptualized as adjunctive and symptom-targeted rather than curative or disorder-modifying.

Across the included studies, the concept of *brief hospitalization* was not operationalized uniformly. Reported durations ranged from overnight admission or stays of less than 48 hours in structured emergency pathways to approximately five days in specialized crisis-intervention units. Despite this variability, a common objective emerged: stabilization of acute risk, formulation of the crisis, reinforcement of coping strategies, and rapid transition toward outpatient care. Similarly, *crisis intervention* generally referred to time-limited, goal-oriented interventions focused on safety planning, emotional stabilization, and linkage to follow-up services rather than extended inpatient treatment.

### 3.5 Quality appraisal

Methodological quality varied across the included studies. While some recommendations were supported by empirical data, including controlled or naturalistic investigations, other conclusions were derived from clinical guidelines, expert consensus, or observational reports (23, 25). This heterogeneity reflects the broader challenge of conducting high-quality interventional research in emergency psychiatric populations with BPD.

The limited number of eligible studies (n = 12) underscores a relative paucity of empirical evidence specifically addressing emergency decision-making in BPD. This restricted evidence base limits the generalizability of findings across emergency settings and healthcare systems, particularly given variations in service organization, admission practices, and access to specialized outpatient care. Consequently, many recommendations remain informed by extrapolation from broader suicide prevention literature or expert consensus. These findings highlight the need for further prospective and comparative research examining hospitalization strategies, crisis intervention models, and pharmacological practices in emergency settings for this population.

Importantly, much of the available literature consisted of observational or expert-driven publications rather than controlled prospective studies. Consequently, several conclusions should be interpreted as practice-informed recommendations rather than high-certainty evidence.

## 4 Discussion

This review highlights three central considerations when evaluating patients with BPD in emergency settings: the imperative to conduct a structured and contextualized suicide risk assessment capable of distinguishing acute suicidal escalation from chronic vulnerability while contextualizing the crisis; the preferential orientation toward brief hospitalization when acute risk is elevated; and the limited role of pharmacotherapy, aside from atypical antipsychotics, in the management of acute agitation.

### 4.1 Suicide risk assessment in a context of chronic vulnerability

Given that suicide represents the leading cause of excess mortality among individuals with BPD compared to the general population, the emphasis on systematic suicide risk assessment is unsurprising and clinically indispensable (7, 15, 30, 31). However, two major challenges must be underscored. First, distinguishing imminent suicidal behavior from the background of chronic suicidal ideation and recurrent self-harm characteristic of BPD remains clinically complex (32, 33). Acute risk may fluctuate rapidly, often in response to interpersonal triggers, and standard risk assessment frameworks may inadequately capture this dynamic instability. Second, existing emergency care systems appear insufficiently protective: notably, approximately one quarter of individuals with BPD who died by suicide had presented to an emergency department within the six weeks preceding their death (15). This finding calls into question the effectiveness of current assessment and disposition practices.

Several avenues may improve clinical outcomes. Enhanced identification of BPD in emergency departments, including the potential use of validated self-report screening tools, may facilitate earlier recognition and more tailored interventions (34). Addressing stigma toward individuals with personality disorders is equally critical, as negative clinician attitudes may influence both risk perception and disposition decisions (35, 36). Educational interventions and structured training programs for emergency clinicians have demonstrated promise in improving attitudes and competencies (37, 38). Finally, the development and implementation of specialized crisis-oriented interventions (either embedded within emergency departments or structured as short-term crisis programs) may provide a more coherent and therapeutic response to acute dysregulation (23, 39–42).

Among available psychotherapeutic approaches, dialectical behavior therapy (DBT) or DBT-brief interventions remains one of the most empirically supported interventions for reducing self-harm behaviors and improving emotional regulation in BPD, reinforcing the importance of rapid referral pathways from emergency settings toward structured outpatient treatment (27). Brief telephone coaching or crisis-oriented DBT-informed interventions may also represent useful adjuncts to reduce recurrent emergency presentations and reinforce continuity of care.

### 4.2 The role of brief hospitalization: substitute or strategic intervention?

In downstream decision-making, brief hospitalization is a pragmatic strategy for addressing acute emotional dysregulation in a contained, therapeutically structured environment (9, 10, 20–24, 27). Unlike prolonged inpatient admission, time-limited crisis hospitalization aims to stabilize acute symptoms, reinforce coping strategies, and facilitate transition to outpatient care without favorizing dependency or regression (13). Evidence suggests that structured short-term crisis interventions (such as five-day crisis hospitalization) may reduce subsequent emergency visits and rehospitalizations within three months (23).

The role of brief hospitalization, however, may be interpreted in two distinct ways. On the one hand, it may serve as a substitute for what should ideally be readily accessible: specialized outpatient psychotherapeutic services for BPD (31). From this perspective, strengthening ambulatory infrastructure and ensuring equitable geographic access to evidence-based treatments could reduce reliance on emergency care and diminish the centrality of hospital-based care. On the other hand, some authors argue that the naturalistic course of BPD symptoms often shows gradual improvement over time, and that maintaining highly specialized teams in all regions is operationally challenging (18, 43, 44). In this view, intermittent, structured, and patient-centered crisis interventions may constitute a realistic and acceptable model of care aligned with patient expectations (45).

These divergent interpretations reflect broader systemic tensions between ideal models of continuous specialized outpatient care and pragmatic adaptations within resource-constrained healthcare systems.

### 4.3 Pharmacotherapy in the emergency context: prudence and system constraints

Consistent with most clinical guidelines and supported by meta-analytic caution regarding the efficacy of psychopharmacology in BPD, this review reinforces the limited role of medications in emergency management (46). Pharmacotherapy should not be initiated routinely during emergency visits, particularly for long-term symptom modification. The evidence base for psychotropic use in BPD remains modest, with many studies characterized by small sample sizes, exploratory designs, and, in some cases, the absence of control groups.

Nonetheless, emergency clinicians often operate within systemic constraints. In contexts where rapid access to specialized outpatient interventions is unavailable, pharmacological options may be perceived as one of the few immediately actionable interventions. Furthermore, the high prevalence of psychiatric comorbidities in BPD (including mood and substance use disorders) naturally increases prescribing patterns, sometimes blurring the distinction between treatment of comorbid conditions and attempts to modulate core personality pathology (4).

Within this limited pharmacological framework, the literature supports the targeted use of atypical antipsychotics for acute agitation (21, 26, 28, 29). Emergency departments should therefore consider structured protocols for agitation management, favoring atypical antipsychotics while avoiding combinations such as first-generation antipsychotics with benzodiazepines due to risks of dystonia and respiratory depression (21). Importantly, such interventions should remain symptom-focused and time-limited rather than construed as disease-modifying treatments.

### 4.4 Limitations

The limited number of eligible studies underscores the relative lack of empirical research specifically addressing the emergency management of BPD. Several recurring concepts (such as “brief hospitalization”) were frequently invoked but insufficiently operationalized or standardized across studies, limiting interpretability and generalizability. Methodological quality was often modest, particularly in studies examining pharmacological interventions, which were largely exploratory and underpowered. Furthermore, the small number of included studies constrained the ability to draw broadly generalizable conclusions and highlights the need for additional multicenter and prospective investigations across diverse emergency care contexts. The absence of standardized definitions for concepts such as brief hospitalization and crisis intervention limited direct comparison across studies. Consequently, findings should be interpreted as reflecting a family of related approaches rather than a single, clearly defined intervention model.

The heterogeneity of study designs further complicated synthesis, as included articles differed substantially in methodology, objectives, populations, and outcome measures. As a result, findings should primarily be interpreted as mapping current clinical approaches rather than establishing definitive evidence-based standards. Additionally, restricting inclusion to studies conducted in Western healthcare systems may limit the applicability of findings to non-Western cultural and organizational contexts. This reflects a broader limitation within psychiatric and psychological research, where evidence is disproportionately derived from Western populations. Because our search was limited to Western healthcare systems, findings may not be directly transferable to non-Western cultural, organizational, and legal contexts, including those found in Asia, the Middle East, Africa, and Latin America. Future cross-cultural investigations are needed to determine whether the clinical pathways and hospitalization practices identified in this review generalize across different healthcare models and cultural understandings of personality pathology.

These limitations constrain the strength of the conclusions that can be drawn and highlight the urgent need for prospective, comparative, and implementation-focused research in emergency psychiatric settings. Future investigations should aim to clarify optimal duration and structure of crisis hospitalization, evaluate structured risk-assessment models adapted to chronic suicidality, and examine integrated pathways linking emergency care to specialized outpatient services. Prospective interventional research in this population may also be constrained by ethical challenges related to suicidality, crisis instability, and fluctuating decisional capacity during emergency presentations.

### 4.5 Recommendations for practice

Based on the findings of this review, several practice-oriented implications can be proposed for emergency psychiatric services managing patients with BPD. First, suicide risk assessment should be both structured and formulation-driven. Clinicians should systematically evaluate established acute and chronic risk factors while simultaneously contextualizing the crisis within interpersonal triggers, treatment discontinuities, and affective dysregulation patterns (20, 21, 32, 33, 47). Risk stratification should not rely solely on static indicators but integrate dynamic clinical judgment tailored to the fluctuating course characteristic of BPD.

Second, emergency departments should develop clear, standardized care pathways for patients with BPD. When hospitalization is indicated due to elevated suicide risk, danger to others, severe symptom exacerbation, impaired self-care, or treatment non-adherence, brief and goal-oriented crisis hospitalization should be prioritized over prolonged admissions (9, 10, 23, 24, 27). Such admissions should include clearly defined therapeutic objectives, early discharge planning, and structured linkage to outpatient follow-up. Whenever possible, emergency services should collaborate with specialized outpatient programs to ensure rapid continuity of care (28, 39–42). In regions where such services are scarce, investment in crisis-oriented interventions embedded within emergency settings may represent a pragmatic and clinically sound alternative.

Third, emergency teams should implement structured protocols for agitation management that prioritize atypical antipsychotics in acute situations while avoiding pharmacological strategies associated with higher adverse-event risk (21, 23–25). Initiation of long-term psychopharmacological regimens in the emergency context should generally be avoided, unless clearly indicated for comorbid psychiatric conditions (4, 9, 43). Medication use should remain symptom-targeted and time-limited, embedded within a broader psychosocial plan.

Finally, emergency systems should prioritize workforce training and stigma reduction initiatives (35–38). Enhancing clinicians’ competencies in BPD assessment, crisis formulation, and therapeutic communication may improve both patient experience and disposition decisions. Integrating screening tools to facilitate earlier identification of BPD presentations may further refine triage and intervention planning (34). Emergency departments are also inherently high-pressure environments, and the presence of complex psychiatric and forensic presentations may increase the risk of incomplete or inaccurate clinical documentation, potentially affecting clinical decision-making, continuity of care, and patient safety (48). Collectively, these recommendations aim to reconcile patient safety, clinical effectiveness, and system feasibility within the high-pressure environment of emergency psychiatric care.

## 5 Conclusion

Encounters between emergency clinicians and individuals living with BPD are frequent and carry a dual significance: they are moments of acute vulnerability, but also opportunities to alter trajectories too often marked by recurrent crises and suicidal behavior. These encounters are inherently complex. Many individuals with BPD present with histories of trauma and disrupted attachment, and may express intense emotional dysregulation, fear of abandonment, and interpersonal sensitivity within the overstimulating and time-pressured environment of the emergency department. The setting itself (crowded, noisy, and oriented toward rapid triage) can amplify distress and reinforce feelings of invalidation.

This complexity is not solely patient-related. It also reflects systemic and relational dimensions. Healthcare providers may hold consciously or unconsciously pessimistic attitudes toward BPD, shaped by repeated crisis presentations and the chronicity of symptoms. At the organizational level, emergency departments are often equipped with tools that are imperfectly aligned with the needs of this population. Psychiatric hospitalization may be available, but not always therapeutically structured for personality pathology, while specialized outpatient psychotherapies such as DBT may remain difficult to access. In such contexts, pharmacological management can become a default response, even when it does not address the disorder’s core features.

This review supports several pragmatic clinical orientations: structured and contextualized suicide risk assessment, brief and goal-directed hospitalization when acute risk is elevated, cautious and symptom-targeted pharmacological use, and stronger integration between emergency and outpatient services. At the same time, the findings underscore broader tensions inherent to emergency psychiatric care, including how clinicians balance acute safety concerns, therapeutic engagement, guideline-based practice, and real-world resource limitations within highly pressured clinical environments.

While further research may clarify optimal crisis interventions and system models, structural reflection remains indispensable. Each healthcare sector must examine how care for individuals with BPD is organized across the continuum, from emergency triage to sustained outpatient follow-up. Without deliberate attention to service design, emergency departments risk functioning as revolving doors rather than therapeutic turning points. The challenge is therefore not only clinical but organizational: to create care pathways that protect life while preserving dignity, therapeutic alliance, and opportunities for long-term stabilization.

Several specific research questions emerge from the current evidence gaps. First, what is the comparative effectiveness of different brief-hospitalization models (e.g., ≤24 hours, 48–72 hours, and approximately five days) in reducing subsequent emergency department presentations, suicide attempts, and rehospitalizations? Second, can existing suicide-risk assessment instruments be adapted and validated for the chronic but fluctuating suicidality characteristic of BPD, thereby improving discrimination between baseline vulnerability and acute escalation? Third, do integrated emergency-to-outpatient pathways, including rapid-access DBT-informed interventions or crisis follow-up clinics, improve treatment engagement and reduce subsequent service utilization compared with usual care? Finally, implementation studies are needed to determine how emergency services can effectively incorporate structured BPD-specific pathways within routine clinical practice.
