# Supplementary material for: Treatment of patients with borderline personality disorder in the emergency room? A scoping review
Source: Front Psychiatry. 2026 Jul 8;17:1820717. doi: 10.3389/fpsyt.2026.1820717 (PMC13390497; doi:10.3389/fpsyt.2026.1820717)
Supplement: Supplementary file 1 [file Table1.docx]

Search Strategies

**BPD – Emergency Settings
Search Strategy
Revision and Update – December 2025**

**#1 PUBMED**

("Personality Disorders"[Mesh:NoExp] OR "Borderline Personality Disorder"[Mesh] OR "Personality disorder*"[TIAB] OR "borderline personality"[TIAB:~2] OR "borderline personalities"[TIAB:~2] OR "borderline state"[TIAB:~2] OR "borderline states"[TIAB:~2]) **AND** ("Emergency Services, Psychiatric"[Mesh] OR "Emergency Service, Hospital" [Mesh:NoExp] OR "Emergency Medical Services"[Mesh] OR Emergency[TIAB] OR emergencies[TIAB])

Filters: English, French, from 2000 - 2025

**= 529 results(December 31^st^ 2025)**

**#2 EMBASE**1. *personality disorder/ or *borderline state/

2. ("Personality disorder*" or (borderline adj2 (personalit* OR state*))).ti,ab.

3. 1 or 2

4. emergency ward/

5. emergency health service/ or hospital emergency service/ or psychiatric emergency service/

6. (Emergency or emergencies).ti,ab.

7. 4 or 5 or 6

8. 3 and 7

9. limit 8 to ((english or french) and yr="2000 - 2025" and (article or article in press or books or chapter or editorial or erratum or letter or note or "review"))

**= 521 results (December 31^st^ 2025)**

**#3 PSYCINFO**

1. personality disorders/ or borderline personality disorder/

2. ("Personality disorder*" or (borderline adj2 (personalit* OR state*))).ti,ab.

3. 1 or 2

4. emergency services/

5. (Emergency or emergencies).ti,ab.

6. 4 or 5

7. 3 and 6

8. limit 7 to (("0100 journal" or "0110 peer-reviewed journal" or "0120 non-peer-reviewed journal" or "0130 peer-reviewed status unknown" or "0200 book" or "0240 authored book" or "0280 edited book") and (english or french) and yr="2000 - 2025")

**= 424 results (December 31^st^ 2025)**

**#4 CINAHL**

S7 S3 AND S6 Limiters - Published Date: 20000101-20251231; Language: English, French

S6 S4 OR S5

S5 Emergency or emergencies

S4 (MH "Emergency Service") OR (MH "Emergency Medical Services") OR (MH "Emergency Services, Psychiatric")

S3 S1 OR S2

S2 "Personality disorder*" OR (borderline N2 (personalit* OR state*))

S1 (MH "Personality Disorders") OR (MH "Borderline Personality Disorder")

**= 257 results (December 31^st^ 2025)**

**Total number of results: 1,731
Total number of duplicates identified at import (title, year): 1,352**

**TOTAL: 379**
